# Molecular docking analysis of phyto-constituents from Cannabis sativa with pfDHFR

**DOI:** 10.6026/97320630014574

**Published:** 2018-12-27

**Authors:** Temitope.I David, Niyi.S Adelakun, Olaposi.I Omotuyi, Damilohun.S Metibemu, Oluwafemi.E Ekun, Gabriel.O eniafe, Olumide.K Inyang, Bamidele Adewumi, Ojochenemi.A Enejoh, Raymond.T Owolabi, Eunice. I Oribamise

**Affiliations:** 1 Centre for Biocomputing and Drug Development, AdekunleAjasin University, AkungbaAkoko, Ondo State, Nigeria; 2 Department of Biochemistry, Adekunle Ajasin University, Akungba Akoko, Ondo State, Nigeria

**Keywords:** Molecular docking, rule of five, pfDHR-TS, *Cannabis sativa*, glide

## Abstract

Available antimalarial drugs have been associated with numerous side effects, which include skin rashes and myelo-suppression. Therefore, it is of interest to explore compounds from natural source having drug-like properties without side effect. This study focuses on the screening of compounds from *Cannabis sativa* against malaria *Plasmodium falciparum* dihydrofolate reductase for antimalarial properties using Glide (Schrodinger maestro 2018-1). The result showed that phytochemicals from *Cannabis sativa* binds with a higher affinity and lower free energy than the standard ligand with isovitexin and vitexin having a glide score of -11.485 and -10.601 respectively, sophoroside has a glide score of -9.711 which is lower than the cycloguanil (co-crystallized ligand) having a glide score of -6.908. This result gives new perception to the use of Cannabis sativa as antimicrobial agent.

## Background

The prevalence of Malaria has been a major cause of mortality in infants and adults globally 1[1]. Research has estimated infections ranging from 300-500 million of human per year globally and deaths recorded are up to 2million annually 2. Protozoan of the genus plasmodium is the major antecedent of the infection and can be siphoned to humans through the saliva of anopheles mosquito 1[2]. Among the species of plasmodium, Plasmodium falciparum is the major cause of mortality and Plasmodium vivax is the mainspring of illness that is associated with malaria, with 2000 cases imported into the UK per year 2. Therefore, it is of interest to develop compounds with improved efficacy to combat malaria.

Myriad of antimalarial drugs have been developed which includes chloroquine, quinolines, antifolates, hydroxyl-naphtha-quinones and artemether 3. Antifolates have been widely used as a potent antimalarial drug with their effectiveness being encumbered by rapid upsurge of resistance at the active site of dihydrofolate reductase 2-4. Antifolate inhibitors target one of the essential pathways for the survival of malaria parasite known as the folate metabolism 3. Two crucial enzymes are being inhibited by antifolates in folate metabolism, which is known as dihydrofolate reductase-thymidinesynthase (DHFR-TS) and dihydropteroate synthase (DHPS). Pyrimethamine, dapsoneproguanil inhibit DHFR and sulfadoxine inhibit DHPS. These enzymes are crucially involved in the de-novo biosynthesis of folate, which is needed for the biosynthesis of purines and pyrimidines 2,3. Therefore, its inhibition impedes the synthesis of essential metabolites needed for the production of nucleotides and proteins.

**Figure 1:**
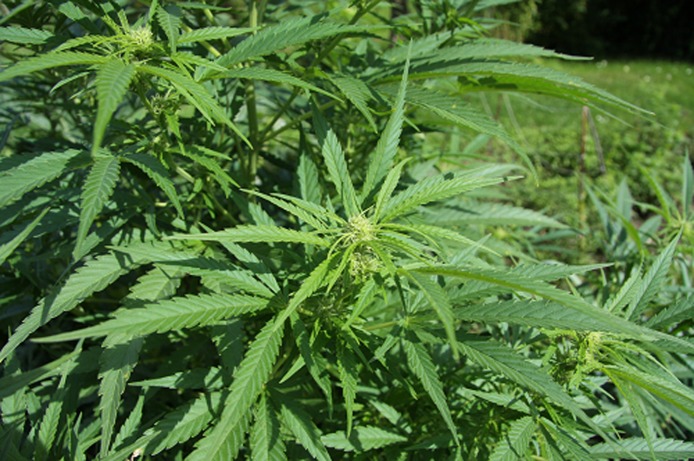
A picture of Cannabis sativa is given 21.

Presently, dihydrofolate inhibitors have been developed for the control of malaria. Still, the downside of their use is the ability of Plasmodium falciparum dihydrofolate reductase to develop resistance to these drugs. This occur as a result of mutation on the amino acid residue at the active site of the enzyme leading to single, double, triple and quadruple mutation [5,6]. Mutation decreases the binding affinity of the inhibitors, thereby decreasing the efficacy of the drug 5. Manifold side effects have been reported to be associated with the use of pfDHFR-TS such as proguanil and pyrimethamine which cause gastro-intestinal upsets, skin rashes and myelo-suppression 2. Therefore, it is essential to develop new antimalarial drug with high efficacy and insignificant side effect as a result of reported mortality, morbidity and spread of malaria with the use of pre-existing drugs and their side effects. Cannabis sativa has been reported to contain more than 540 in total 20, and they are rich in compounds such ascannabinoids and noncannabinoids, flavonoids, flavonoidglucosides andsophoroside 7,8. Flavonoids are polyphoniccompounds, known to exhibit antimalarial properties. In this study, phyto-constituent from Cannabis sativaexhibit antimalarial properties against pfDHFR-TS with isovitexin, vitexin and sophoroside having a binding score of - 11.485, -10.601 and -9.711 respectively. A picture of Cannabis is given in Figure 1.

## Methodology

Glide tool from Schrodinger molecular drug discovery suite (version 2018-1) was used in this research work.

### Ligand Selection and Preparation:

Eighty-one Characterized phyto-chemicals of Cannabis sativa used in this study were obtained from published literatures and they were used in the generation of library of compounds in this study. The library of compounds obtained was downloaded from NCBI PubChem database (https://www.ncbi.nlm.nih.gov/pccompound) in 2d (sdf) format. The phyto-chemicals generated were prepared using the Ligprep interface in Schrodinger 10 with an OPLS3 force field, at pH 6 ± 1 using Epik 11. Desalt and generate tautomers were also selected on the ligprep interface and the stereoisomer computation was left at retain specific chiralities (vary other chiral centers) and to generate at most 32 per ligand.

### Protein Selection and Preparation:

Crystallized three-dimensional structure of the target protein which is Plasmodium falciparum dihydrofolate reductase-thymidine synthase (pfDHFR-TS) in complex with co-crystallized ligand:cycloguanil, PDB ID:3UM8 12 was retrieved from protein data bank(http://www.rcsb.org/pdb/home/home.do). Its selection is as a result of the presence of an inhibitor ligand at the active site of the protein. The crystallized 3d structure was viewed with maestro 11.5 interface and prepared with protein preparation wizard at a pH of 6 ± 1. Also, water molecules and other interfering ligands were removed from the protein during the preparation process.

### Receptor Grid generation:

The Receptor grid defines the area of interaction between the protein and the ligand. This was carried out with the receptor grid generation tool in maestro 11.5 which defines the area around the active site in term of co-ordinates x, y and z. The receptor grid box resolution was centered at coordinates 29.72, 5.25 and 58.31 corresponding to x, y and z-axis, respectively.

### Molecular Docking using Glide:

The Docking analysis was accomplished using Glide tool on maestro 11.5 13,14. The prepared library of ligands (phytochemicals) were docked into the active site of the target protein (3um8) using the standard precision algorithm (SP) with the ligand sampling treated as flexible then followed by extra precision (XP) with ligand sampling as none refine only. Docking analysis was first carried out on the co-crystallized ligand to determine its binding affinity at the active site of the target protein prior to docking of the libraries of compounds. The ligand interaction tool was used to view the interaction diagram of the ligands with the residues at the active site of the target protein.

### ADMET/Tox Screening:

The Hit compounds were further subjected to Absorption, Distribution, Metabolism, Excretion and Toxicity using the Qikprop tool 15.

### Validation of Molecular docking Result:

**Figure 2:**
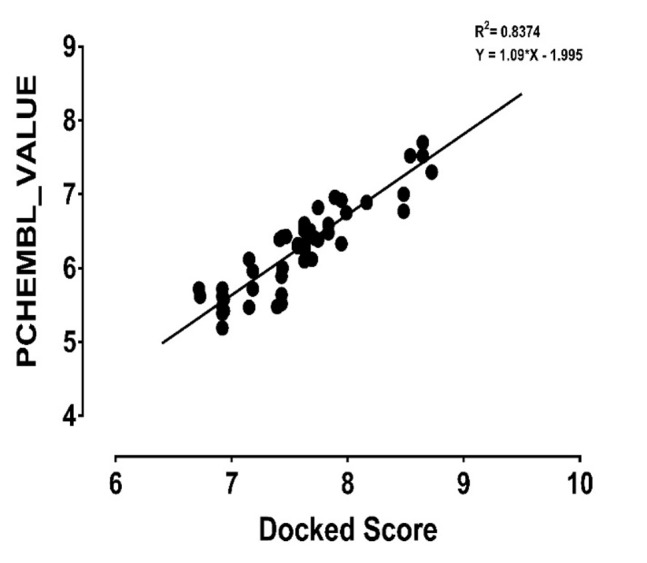
Showing the correlation graph between the experimentally determined pIC50 of pfDHFR andtheir docked scores. r^2^ (correlation of determination) of 0.8374 was observed which denotes that Docking experiment can reproduce the experimentally determined values of the inhibitors

The docking protocol employed was validated by blasting the fasta sequence of co-crystalized target protein with PDB ID: 3um8with the CHEMBL database server (www.ebi.ac.uk/chembl/) 16. From the search result, bioactivities of compounds from the dataset with IC50 value of 341 and inhibition 719 of were downloaded with the canonical smiles of the compounds. The bioactivity files were pasted into Microsoft excel sheet (Microsoft office suite 2016) to display the properties of the downloaded compounds. The bioactivities were sorted out to delete missing or misplaced data to remain a total of 50 valid data. This valid data were converted to 2d (sdf) form using data warrior software version 2. The ligands were imported into schrodinger maestro 11.5 (2018-1) and prepared using the ligprep tool at a pH of 6 ± 1 and a forcefield of OPLS3.The prepared ligands were docked using glide into the target protein receptor using XP precision algorithm. A graph of the correlation coefficient graph of the XP docking score of selected 50 compounds and PCHEMBL_VALUE (experimentally determined) was plotted as shown in Figure 2. The r2 spearman correlation was generated between the PCHEMBL_VALUE and the XP docking scores of the compounds.

## Results and Discussion

Molecular interaction between protein and ligand predicts the binding conformation or pose of the ligand bounded to the protein, which can be quantified, based on the shape and electrostatic interaction between the ligand and protein 17. The totality of interaction observed is approximated to be the docking score of the ligand into the binding pocket of the protein 17. Docking score is expressed in negative value of energy in Kcal/mol where the lower the negative total energy E, the stronger the interaction between the ligands and the protein 18. The Library of compounds generated was subjected to docking experiment to determine compounds with high binding energy than co-crystallized ligand of pfDHFRTS. Docking approach predicts the best binding conformation of the compounds at the binding pocket of the protein and the interaction between the ligand and the residues at the active site of the enzyme. The docking result shows the binding energy of the 12 hit compounds of Cannabis sativa out of 81-screened library of compounds that was retrieved from NCBI database against pfDHRTS. The XP precision used gives a more accurate docking result. The docking result and ADME screening of the phyto-chemical, cocrystallized ligand and a standard inhibitor pyri-methamine was shown in Table 1.

### Interaction Profiling of pfDHFR-TS Inhibitors:

**Figure 3:**
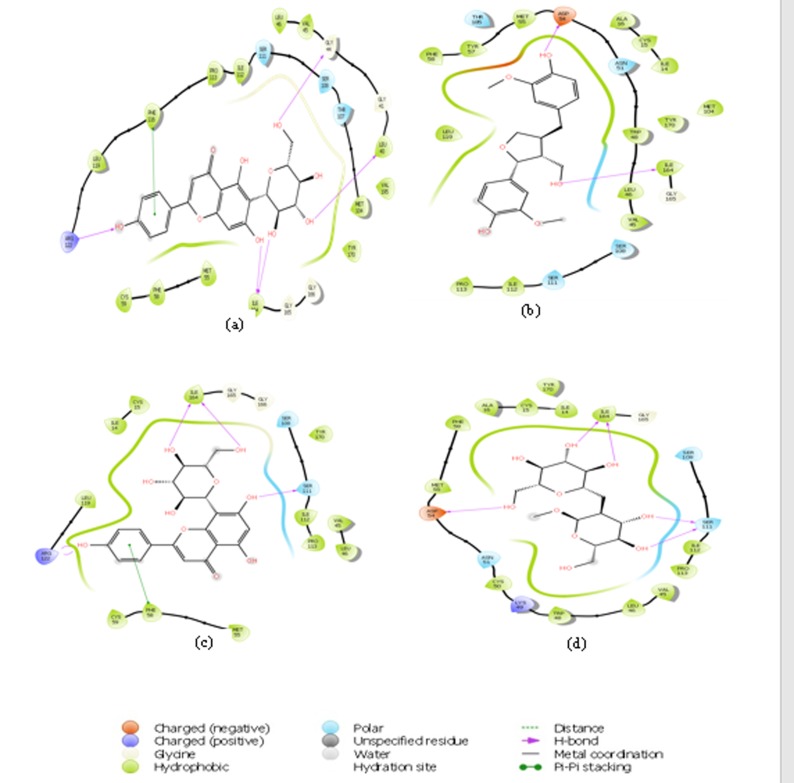
2D stick diagram of (a) isovitexin; (b) lariciresinol; (c) vifexin; (d) sophoroside illustrating hydrogen bonds and pi-pi stacking formed with the aminoacid residues at the binding pocket of pfDHFR-TS.

The mechanism of interaction of potential inhibitors of plasmodium dihydrofolate reductase has been exclusively studied both in wild type and mutant type of the protein. Inhibition of the enzyme is dependent on the formation of different type bonds between the amino acid residue at the active site and the ligand. The elimination of toxic effect that might result is due to the specificity of the compounds to interact with the amino acid residues at the active site of pfDHFR-TS. The amino acid residue of human DHFR differs from that of pfDHFR-TS by the replacement of residuePhe31, Gln35, and Asn64 in human with Met55, Cys /Arg59, and Phe116 in pfDHFR-TS 5. This residue affects the binding of compounds around the vicinity of the conserved Arg122 5. For inhibition to occur, interaction is needed on some key amino acid residue; Asp54, Asn/Ser108, Ileu/Leu164 and Ile14at the active site of the enzyme 19. Figure 3 shows that Isovitexin forms a pi-pi stacking using it phenyl ring with Phe116, while a proton is being donated from conserve Arg122 at the active site to the hydroxyl group attached at C31. It was observed that hydroxyl group attached at C19 and C11 donated two protons to Ile164. Sophoroside and lariciresinol adopt similar pattern of interaction in which a hydrogen bond is formed by donating a proton to Asp54 and also to Ile164 residue at the binding pocket of the protein. Vitexin interacted with the conserve arginine 122 in a similar manner with its hydroxy group at C31 and also forms two hydrogen bonds with Ile 164 by accepting two protons from C17 and C14.

### ADME/TOX and Rule of Five (ROF)

#### ADME Screening

The screening of compounds using Absorption, distribution, metabolism and elimination (ADME) describes the efficacy, ability of the compounds to reach its target protein and to be easily eliminated from the body. Computational approach to drug design help to screen large database of compounds in order to reduce the cost and time of subjecting diverse compound into molecular analysis. The Lipinski's rule of five (ROF) enlist some criteria that is needed for a compound to be considered to be drug like in nature, this criterion includes a molecular weight that is less than 500Da (<500Da), hydrogen bond donors that is less or equal to 5, hydrogen bond acceptors that is less or equal to 10 and octanolwater partition coefficient (logP) that is less than 5(<5). Therefore, compounds that are coherent with this rule are considered to be drug-like in nature. From Table 1 above, Compounds such as Cannabitriol, lariciresinol and sophoroside from Cannabis sativa are in accordance with this rule and also with high and medium Human oral absorption (HOA). Therefore, they can be considered to be drug and some can be modified as potent inhibitor of pfDHFR-TS, which can be subjected to further studies.

## Conclusion

This study shows the binding ability of a library of compounds generated from Cannabis sativa as potential inhibitors of plasmodium dihydrofolate reductase. Isovitexin has a higher binding affinity with pFDHFR-TS, interacting with amino acid residue via hydrogen bonds and pi-pi stacking. This data provides a new perception and view into pharmacological use of Cannabis sativa for malaria.

## Figures and Tables

**Table 1:** Docking results with pharmacological properties.

S.No	Entry Name	Glide Gscore (Kcal/mol)	Dock score (Kcal/mol)	ROF Violation	HOA	MW	QlogKhsa
1	isovitexin	-11.489	-11.485	1	Medium	432.383	-0.669
2	vitexin	-10.604	-10.601	1	Medium	432.383	-0.667
3	sophoroside	-9.711	-9.711	1	Medium	354.353	-1.347
4	cannflavinA	-9.513	-9.513	1	Low	436.504	1.119
5	secoisolariciresinol	-9.205	-9.205	0	Medium	362.422	-0.226
6	lariciresinol	-8.84	-8.84	0	High	360.406	0.051
7	quercetin	-8.423	-8.421	0	Medium	302.24	-0.349
8	cannabitriol	-8.415	-8.415	0	High	346.466	0.537
9	kaempferol	-8.047	-8.045	0	High	286.24	-0.195
10	luteolin	-7.791	-7.788	0	High	286.24	-0.194
11	catechin	-7.328	-7.328	0	Medium	290.272	-0.422
12	chrysin	-6.976	-6.972	0	High	254.242	0.128
13	cycloguanil(co-crystallized)	-6.908	-6.908	1	Medium	261.797	-0.387
14	pyrimethamine	-6.957	-6.957	0	High	248.714	-0.265
